# Pathway analysis of expression-related SNPs on genome-wide association study of basal cell carcinoma

**DOI:** 10.18632/oncotarget.9212

**Published:** 2016-05-06

**Authors:** Xin Li, Liming Liang, Immaculata De Vivo, Jean Y. Tang, Jiali Han

**Affiliations:** ^1^ Department of Epidemiology, Harvard T.H. Chan School of Public Health, Boston, MA, USA; ^2^ Channing Division of Network Medicine, Department of Medicine, Brigham and Women's Hospital and Harvard Medical School, Boston, MA, USA; ^3^ Department of Dermatology, Stanford University School of Medicine, Redwood City, CA, USA; ^4^ Department of Epidemiology, Fairbanks School of Public Health, Indiana University, and Indiana University Melvin and Bren Simon Cancer Center, Indianapolis, IN, USA

**Keywords:** expression-related SNPs (eSNPs), pathway analysis, basal cell carcinoma (BCC), genome-wide association study (GWAS), pathway databases

## Abstract

Genome-wide association studies (GWASs) have primarily focused on the association between individual genetic markers and risk of disease. We applied a novel approach that integrates skin expression-related single-nucleotide polymorphisms (eSNPs) and pathway analysis for GWAS of basal cell carcinoma (BCC) to identify potential novel biological pathways. We evaluated the associations between 70,932 skin eSNPs and risk of BCC among 2,323 cases and 7,275 controls of European ancestry, and then assigned them to the pathways defined by KEGG, GO, and BioCarta databases. Three KEGG pathways (colorectal cancer, actin cytoskeleton, and BCC), two GO pathways (cellular component disassembly in apoptosis, and nucleus organization), and four BioCarta pathways (Ras signaling, T cell receptor signaling, natural killer cell-mediated cytotoxicity, and links between Pyk2 and Map Kinases) showed significant association with BCC risk with *p-value*<0.05 and FDR<0.2. These pathways also ranked at top in sensitivity analyses. Two positive controls in KEGG, the hedgehog pathway and the BCC pathway, showed significant association with BCC risk in both main and sensitivity analyses. Our results indicate that SNPs that are undetectable by conventional GWASs are significantly associated with BCC when tested as pathways. Biological studies of these gene groups suggest their potential roles in the etiology of BCC.

## INTRODUCTION

Basal cell carcinoma (BCC), a major type of non-melanoma skin cancer, is the most common malignancy among populations of European ancestry [1–3]. Though rarely fatal, the tumor may be locally invasive and cause clinically significant destruction of surrounding tissue if not treated adequately [4, 5]. In addition, subsequent skin cancers and other malignancies are more common among BCC patients in comparison to the general population [6].

Both environmental and genetic factors contribute to the genesis of BCC. Though exposure to ultraviolet (UV) radiation is generally accepted as the most important environmental risk factor for BCC, other known risk factors include family history of skin cancer and pigmentary characteristics, such as fair complexion, red or blond hair, and light eye color [7–9]. Most recently, genome-wide association studies (GWAS) have identified several genetic loci (including 1p36, 1q42, 5p15, 7q32, and 9q21, among others) associated with risk of BCC [10–12]. Despite the advances that have been made in understanding the etiology of BCC, the genetics of this complex disease is still largely unknown.

Although GWASs have revolutionized our ability to identify disease susceptibility loci or markers associated with them, they usually yield only the most significant SNPs, and the percentage of genetic variation explained by GWAS signals has generally been modest [13, 14]. One of the potential explanations for this “missing” heritability is that most common DNA variants with moderate effect size have not yet been identified by GWAS because of a lack of power [15]. Given this limitation of conventional association analysis, new approaches are emerging to enhance the information extracted from current GWAS data. Pathway analysis, which jointly considers multiple variants with moderate signals in related genes, is a good complement to single-locus GWAS [16]. There is growing evidence that complex molecular networks and cellular pathways are often involved in disease susceptibility and disease progression [17, 18]. Thus, by taking into account prior biological knowledge about genes and pathways, we may have a better chance to identify disease-relevant loci [19], even though the signals individually do not meet the GWAS significance threshold [16].

Borrowing ideas from gene set enrichment analysis (GSEA) in the gene expression microarray field [20], Wang *et al.* first proposed pathway-based analysis of GWAS data in 2007 [16]. They used SNPs that are physically located in the gene region as the representative SNPs for that particular gene. However, SNPs within a gene region may not be the functional variants of the gene, and a gene may be regulated *in trans* by genetic variants that are physically distant [21]. Having realized this major shortcoming of conventional pathway analysis, as well as the importance of genetic variants that regulate gene transcription in mapping human disease genes [22], Zhong *et al.* suggested integration of expression-related SNPs (eSNPs) into conventional pathway analysis [23]. Two main aspects of this new approach are appealing: first, it further improves the power to detect genetic associations, because eSNPs can be considered functionally relevant variants [24]; secondly, it improves the interpretation of results, because variants that cluster within common biological pathways are taken into account jointly. This method has recently shown its potential strength in the context of type 2 diabetes GWAS [25]; however, applications to cancer have rarely been reported.

In 2012, Zhang *et al*. applied this novel pathway analysis to the GWAS of basal cell carcinoma for the first time [26]. Though that study provided novel insights into the biology underlying BCC, the false discovery rates of the identified pathways are of only marginal significance. Moreover, they used eSNPs discovered in two GWASs of global gene expression in lymphoblastoid cell lines (LCL) [22], which is not a tissue relevant to BCC. Because tissue dependency seems to be an important feature of disease susceptibility variants that regulate gene expression [27], ideally skin eSNPs should be used in BCC studies. Recently, the Multiple Tissue Human Expression Resource (MuTHER) project published detailed genomic and transcriptome data on three disease-relevant tissues (adipose, LCLs, and skin) originating from a cohort of 856 deeply phenotyped twins [28]. In the current study, we conducted a skin eSNPs-integrated pathway analysis for GWAS on BCC using MuTHER resources and sought to provide more insights into the underlying mechanisms of BCC.

## RESULTS

From the MuTHER data, we identified 70,932, 87,481, and 97,903 eSNPs in skin tissue using the threshold of 10^−5^ (main analysis), 5×10^−5^ (sensitivity 1), and 10^−4^ (sensitivity 2) respectively. Among them, 69,988, 86,325, and 96,603 are available in our BCC GWAS, respectively. Because all these eSNPs have MAF >1% and imputation R-square >0.4 in our BCC GWAS, they were used for further analysis.

In our main analysis, 2,049 genes with surrogate eSNPs were assigned to the pathways defined in the KEGG database. Using the cut-off of containing 3 to 200 genes, 143 pathways were tested for their associations with BCC risk using our GWAS data. Eleven pathways reached a nominal *p* value < 0.05, which was 1.54-fold higher than the number expected by chance (0.05×143 = 7.15; this is a conservative estimate, because pathways may be correlated due to overlapping genes, and the effective number should be smaller than 143). Three out of the 11 pathways had a FDR <0.2: the colorectal cancer pathway (p-value<0.00001, FDR =0.005), the regulation of actin cytoskeleton pathway (p-value=0.03, FDR =0.073), and the basal cell carcinoma pathway (p-value=0.002, FDR =0.069). In sensitivity 1 analysis, the numbers of genes that can be represented by eSNPs increased to 2,649 when we used the threshold of 5×10^−5^ for eSNP identification. A total of 151 KEGG pathways that contain between 3 and 200 genes were examined for their associations with BCC. Twelve reached a nominal p<0.05, which was 1.59-fold higher than the number expected by chance. Five out of the 12 pathways had a FDR <0.2. Besides the three that have already been found in the main analysis, the other two pathways are the adherens junction pathway (p-value=0.028, FDR =0.145) and the pancreatic cancer pathway (p-value=0.023, FDR =0.189). In sensitivity 2 analysis, 3,158 genes were included, and 164 KEGG pathways were tested. Fifteen reached a nominal p<0.05, which was 1.83-fold higher than the number expected by chance. Only one out of the 15 pathways had a FDR < 0.2 -- the colorectal cancer pathway (p-value<0.00001, FDR =0.175). In total, five KEGG pathways have shown significant associations with risk of BCC in either main analysis or sensitivity analysis. Results of main and sensitivity analyses for the five significant pathways are listed in Table 1. We also used GO and BioCarta databases for pathway construction. The results are shown in Tables 2 and 3.

**Table 1:** KEGG Pathways with significant enrichment (p<0.05, FDR <0.2) in BCC GWAS & Hedgehog Signaling Pathway

Pathway	Gene count^d^	Main analysis^a^			Sensitivity analysis 1^b^			Sensitivity analysis 2^c^		
		Size^%^	Pathway enrichment p-value^e^	FDR^f^	Size^%^	Pathway enrichment p-value^e^	FDR^f^	Size^%^	Pathway enrichment p-value^e^	FDR^f^
Colorectal Cancer	114	7	***<0.00001***	***0.005***	10	***0.003***	***0.172***	12	***<0.00001***	***0.175***
Regulation of Actin Cytoskeleton	276	14	***0.03***	***0.073***	18	***0.03***	***0.183***	27	0.529	0.952
Basal Cell Carcinoma	73	3	***0.002***	***0.069***	4	***0.001***	***0.169***	4	<0.00001	0.269
Adherens Junction	110	7	0.346	1	10	***0.028***	***0.145***	11	0.02	0.253
Pancreatic Cancer	115	3	0.054	0.163	5	***0.023***	***0.189***	7	<0.00001	0.213
Hedgehog Signaling Pathway	74	3	0.008	0.657	5	0.031	0.464	5	0.036	0.404

a eSNPs were selected at significance level of 10^−5^.

b eSNPs were selected at significance level of 5×10^−5^.

c eSNPs were selected at significance level of 10^−4^.

d The number of genes in the pathway according to the KEGG database.

e &f Based on 1,000 permutations.

f Based on 143, 151, and 164 pathways in main, sensitivity 1, and sensitivity 2, respectively.

% The number of genes that have surrogate eSNPs in the pathway.

**Table 2:** GO Pathways with significant enrichment (p<0.05, FDR <0.2) in BCC GWAS

Pathway^#^	Gene count^d^	Main analysis^a^			Sensitivity analysis 1^b^			Sensitivity analysis 2^c^		
		Size^%^	Pathway enrichment p-value^e^	FDR^f^	Size^%^	Pathway enrichment p-value^e^	FDR^f^	Size^%^	Pathway enrichment p-value^e^	FDR^f^
GO0006921	42	3	***0.007***	***0.179***	4	0.042	0.932	5	0.137	0.941
GO0006997	70	4	***0.025***	***0.120***	5	0.099	0.717	7	0.166	0.902

a eSNPs were selected at significance level of 10^−5^.

b eSNPs were selected at significance level of 5×10^−5^.

c eSNPs were selected at significance level of 10^−4^.

d The number of genes in the pathway according to the GO database.

e&f Based on 1,000 permutations.

f Based on 407, 456, and 506 pathways in main, sensitivity 1, and sensitivity 2, respectively.

# Annotation: GO0006921 – cellular component disassembly involved in apoptosis; GO0006997 – nucleus organization: a process at the cellular level which results in the assembly, arrangement of constituent parts, or disassembly of the nucleus.

% The number of genes that have surrogate eSNPs in the pathway.

**Table 3:** BioCarta Pathways with significant enrichment (p<0.05, FDR <0.2) in BCC GWAS

Pathway^#^	Gene count^d^	Main analysis^a^			Sensitivity analysis 1^b^			Sensitivity analysis 2^c^		
		size	Pathway enrichment p-value	FDR	size	Pathway enrichment p-value	FDR	Size^%^	Pathway enrichment p-value^e^	FDR^f^
rasPathway	23	NA^+^			NA^+^			3	***0.008***	***0.109***
tcrPathway	45	NA^+^			NA^+^			6	***0.014***	***0.189***
nkcellsPathway	20	NA^+^			NA^+^			4	***0.024***	***0.188***
Pyk2Pathway	27	NA^+^			NA^+^			4	***0.048***	***0.199***

a eSNPs were selected at significance level of 10^−5^.

b eSNPs were selected at significance level of 5×10^−5^.

c eSNPs were selected at significance level of 10^−4^.

d The number of genes in the pathway according to the BioCarta database.

e&f Based on 1,000 permutations.

f Based on 60, 71, and 114 pathways in main, sensitivity 1, and sensitivity 2, respectively.

+ These four pathways were not tested in main and sensitivity 1 analyses because their sizes are not between 3 and 200.

# Annotation: rasPathway – Ras signaling pathway; tcrPathway – T cell Receptor signaling pathway; nkcellsPathway -- Ras-Independent pathway in NK cell-mediated cytotoxicity; Pyk2Pathway -- Links between Pyk2 and Map Kinases.

% The number of genes that have surrogate eSNPs in the pathway.

For certain pre-defined pathways identified through the pathway databases, only some of the genes could be represented by eSNPs. Therefore, more attention should be given to the genes and eSNPs that were included in the gene set enrichment analysis rather than to the entire pathway. For significant pathways, we summarized information on such genes and their corresponding eSNPs in Table 4. Because no BioCarta pathway appeared to be significantly associated with BCC risk in main analysis (Table 3), we reported the results of sensitivity analysis 2 for BioCarta in Table 4. On the other hand, some genes belong to more than one of the significant pathways. For example, SOS1 and RAC1 were included in four significant pathways and PIK3R1 and CYCS were in three significant pathways. Nine eSNPs associated with BCC risk at a nominal P < 0.05 are worth noting. These gene-eSNP pairs are CYCS-rs39454 (*P_BCC_* = 0.025), SOS1-rs12473092 (*P_BCC_* = 0.029), ARHGEF7-rs7984371 (*P_BCC_* = 0.039), ITGA2-rs3212544 (*P_BCC_* = 0.040), VCL – rs12360087 (*P_BCC_* = 0.002), BMP2-rs6054443 (*P_BCC_* = 0.0006), BIRC7-rs1075557 (*P_BCC_* = 0.014), PIK3R1-rs9291926 (*P_BCC_* = 0.016), and RAC1-rs2689420 (*P_BCC_* = 0.013).

**Table 4:** Genes and eSNPs in significant pathways identified in main analysis^&^

Pathway database	Pathway	Number of Genes with eSNP	Pathway enrichment *p-value*	FDR	Genes with eSNP	Chr^&&^	Surrogate eSNP^+^	eSNP P_BCC_^#^	Chr_position^##^
KEGG	Colorectal Cancer	7	<0.00001	0.005	BIRC5	17	rs4789559	0.1304	17:76218857
					CYCS	7	rs39454	0.0253	7:25135783
					FZD3	8	rs12678890	0.0746	8:28451002
					FZD8	10	rs11815242	0.1011	10:35995340
					MAPK9	5	rs3812067	0.1035	5:179709154
					SMAD3	15	rs7176870	0.0970	15:67388553
					SOS1	2	rs12473092	0.0291	2:39204040
	Regulation of Actin Cytoskeleton	14	0.03	0.073	ACTG1	17	rs12952655	0.7171	17:80421139
					ARHGEF7	13	rs7984371	0.0385	13:111958666
					BAIAP2	17	rs4969387	0.3086	17:79081724
					C3orf10	3	rs279545	0. 0513	3:9972493
					CYFIP2	5	rs11744003	0.0853	5:156806993
					FGFR4	5	rs422421	0.0994	5:176517326
					GNA12	7	rs7790322	0.0511	7:2830498
					ITGA2	5	rs3212544	0.0404	5:52358887
					ITGAX	16	rs11150612	0.1029	16:31357760
					MYL2	12	rs16941319	0.5931	12:111646853
					PAK2	3	rs7646247	0.4314	3:196519209
					SOS1	2	rs12473092	0.0291	2:39204040
					TIAM1	21	rs2833271	0.2804	21:32487749
					VAV3	1	rs11185131	0.6043	1:108078183
					VCL	10	rs12360087	0.0023	10:76373904
	Basal Cell Carcinoma	3	0.002	0.069	BMP2	20	rs6054443	0.0006	20:6647580
					FZD3	8	rs12678890	0.0746	8:28451002
					FZD8	10	rs11010260	0.0513	10:35995340
GO	GO0006921	3	0.007	0.179	BIRC7	20	rs1075557	0.0143	20:61870465
					CYCS	7	rs39454	0.0253	7:25135783
					DFFB	1	rs4074709	0.8019	1:3796948
	GO0006997	4	0.025	0.120	BIRC7	20	rs1075557	0.0143	20:61870465
					CYCS	7	rs39454	0.0253	7:25135783
					DFFB	1	rs4074709	0.8019	1:3796948
					PML	15	rs11072463	0.1986	15:74303349
BioCarta	rasPathway	3	0.008	0.109	PIK3R1	5	rs9291926	0.0163	5:67599656
					RAC1	7	rs2689420	0.0130	7:6410321
					RALGDS	9	rs482670	0.3617	9:136007358
	tcrPathway	6	0.014	0.189	CALM3	19	rs973679	0.4014	19:47061564
					NFATC2	20	rs231583	0.4901	20:49346881
					NFATC3	16	rs13338993	0.2890	16:67515312
					PIK3R1	5	rs9291926	0.0163	5:67599656
					RAC1	7	rs2689420	0.0130	7:6410321
					SOS1	2	rs12473092	0.0291	2:39204040
	nkcellsPathway	4	0.024	0.188	PIK3R1	5	rs9291926	0.0163	5:67599656
					PTK2B	8	rs472865	0.8824	8:26698471
					RAC1	7	rs2689420	0.0130	7:6410321
					SYK	9	rs914925	0.7664	9:93584793
	Pyk2Pathway	4	0.048	0.199	CALM3	19	rs973679	0.4014	19:47061564
					PTK2B	8	rs472865	0.8824	8:26698471
					RAC1	7	rs2689420	0.0130	7:6410321
					SOS1	2	rs12473092	0.0291	2:39204040

& For the BioCarta database, results of sensitivity analysis 2 are presented in this table, because no significant pathway has been identified in main and sensitivity 1 analysis.

&& Chromosome of genes.

+ If a gene's expression is associated with multiple eSNPs, we used the eSNP that was most significantly associated with BCC risk as the gene's surrogate eSNP.

# P_BCC_ represents P values of the association between eSNPs and risk of BCC.

## Chromosome and position of eSNPs.

Moreover, we chose two established BCC-related pathways in the KEGG database as positive controls – the basal cell carcinoma pathway and the hedgehog signaling pathway. The gene set enrichment p-value for these two pathways reached nominal significance in both the main and sensitivity analyses, though the FDRs of the hedgehog signaling pathway are above 0.2 (Table 1).

## DISCUSSION

Conventional GWASs have primarily focused on the associations between individual genetic markers and risk of diseases. In the current study, we applied a novel approach that integrates skin eSNPs and pathway analysis for GWAS of BCC. Three KEGG pathways (colorectal cancer, regulation of actin cytoskeleton, and basal cell carcinoma), two GO pathways (cellular component disassembly involved in apoptosis, and nucleus organization), and four BioCarta pathways (Ras signaling pathway, T-cell receptor signaling pathway, Ras-independent pathway in natural killer (NK) cell-mediated cytotoxicity, and links between Pyk2 and Map Kinases) showed significant associations with BCC risk. Our results demonstrate that SNPs and genes of moderate effect that are undetectable by conventional GWASs are significantly associated with risk of BCC as groups. These gene sets might be implicated in the etiology of BCC.

Some well-known cancer-related pathways have been mapped in both the colorectal cancer pathway and the BCC pathway in KEGG, including the p53 signaling pathway, the Wnt signaling pathway, the PI3K-Akt signaling pathway, the TGF-β signaling pathway, and other pathways related to cell cycle and survival. Studies have shown that a personal history of non-melanoma skin cancer was significantly associated with a higher risk of other primary cancers [6, 29]. Certain genetic components may act systemically and play a role in both cutaneous and internal carcinogenesis. The actin cytoskeleton pathway mainly regulates cell motility, which is required for many biological processes, such as embryonic morphogenesis, immune surveillance, and tissue repair and regeneration. Aberrant regulation of cell migration drives progression of many diseases, including cancer invasion and metastasis [30, 31]. In the GO database, GO0006921 is defined as the breakdown of structures such as organelles, proteins, or other macromolecular structures during apoptosis; GO0006997 is defined as a process that is carried out at the cellular level that results in the assembly, arrangement of constituent parts, or disassembly of the nucleus, all of which are highly related to cancer development. The RAS signaling pathway is a key regulator of normal cell growth and malignant transformation. Mutations in RAS genes or alterations in upstream or downstream signaling components have been found in most human tumors [32] including basal cell carcinoma, although with a relatively low mutation rate [33]. T cell receptor (TCR) activation promotes a number of signaling cascades that ultimately determine cell survival, proliferation, and differentiation. High levels of intratumor infiltration of T cells is correlated with prolonged survival in cancer patients [34]. NK cells are large granular lymphocytes with natural cytotoxicity against tumor cells [35]. An 11-year follow-up study has shown that low NK cell activity in peripheral blood is associated with increased cancer risk [36].

In the current study, we made a major improvement by using high-quality eSNPs data on disease-relevant tissue. Although detailed gene-expression studies have profiled transcripts and genotyped SNPs across the human genome in several population-based cohorts, gene expression data in skin tissue from a fairly large cohort was not accessible until the publication of the MuTHER project. In that study, the GWAS data and expression data had undergone stringent quality controls before testing the association of expression levels with probabilities of imputed genotypes. Also, skin eSNP identified in the MuTHER study had been replicated in independent cohorts [12]. Other strengths of our study include involvement of multiple pathway databases and design of sensitivity analysis as well as positive controls to validate our findings.

The main limitation of our study is that the proportion of genes that could be represented by eSNPs within a predefined pathway is too small, because only 69,988 SNPs that were significantly associated with expression of 2,049 genes at significance level of 10^−5^ had been included in the main analysis. For example, the colorectal cancer pathway in the KEGG database is composed of 114 genes, whereas only 7 genes (6%) were involved in the gene set enrichment analysis. Specifically, we found that a subgroup of seven genes – BIRC5, CYCS, FZD3, FZD8, MAPK9, SMAD3, and SOS1 – that belong to the KEGG colorectal cancer pathway showed significant association with risk of BCC. Similar conclusions could be drawn for other significant pathways, with the subgroups presented in Table 4. Given that the identified subgroups could hardly represent the original KEGG, GO, and BioCarta pathways, some may argue the necessity of using these pathway resources. However, these pre-defined pathways are important in two ways: on the one hand, they provide us prior knowledge on how to assign genes into different groups in order to conduct a pathway-based analysis; on the other hand, genes have been carefully selected, organized, and mapped in these established pathways based on multiple sources of evidence. With high-quality pre-collected information, we could interpret a gene's role and its relationship with other genes in the same pathway more easily, despite the limited size of identified subgroups.

A further limitation is that no replication was conducted for the identified gene groups, because we used all our BCC GWAS at the discovery stage to maximize statistical power. However, the significant gene groups in the main analysis also ranked top among all pathways being tested in sensitivity analyses. Besides, the positive controls – the Hedgehog signaling pathway and the BCC pathway – were significantly associated with risk of BCC (*p*<0.05) in both main and sensitivity analyses.

We also acknowledge the limitation that the expression data from the MuTHER project might not be very broadly representative as this project only included female participants. Gene expressions differ between females and males, but the vast majority of these differences is attributable to genes that are expressed in reproductive tissues [37]. Though less is known about the sex-specific gene expression pattern in human skin tissue, a study on human blood shows that the genes which express with a significant gender bias tend to locate on the X or Y chromosome [38]. Because only autosomal genes were considered in our study and skin tissue does not belong to reproductive system, using eSNPs identified among female individuals may be acceptable, but not perfect.

Moreover, BCC cases were self-reported without further pathological confirmation in the current study. However, the validity of self-reported BCC in these medically sophisticated populations has been assessed in previous studies [39]. Colditz *et al.* [39] evaluated the validity of self-reported illnesses including skin cancer in the NHS. Among 33 random samples of women who had reported non-melanoma skin cancer, medical records indicated that 30 (91%) had correctly reported their skin cancer. Also, Hunter *et al.* [40] previously examined the risk factors of BCC in the NHS using the self-reported cases. As expected, they found that lighter pigmentation and higher tendency to sunburn were associated with an increased risk of BCC. In addition, using the self-reported BCC cases, our group identified the previously well-documented genetic variant in the MC1R gene as the top risk locus in our GWAS for BCC [12]. These data support the validity of self-report of BCC in our study.

In conclusion, our study identified novel genes and gene sets that may be important for BCC development. Genes with moderate effect that are undetectable in conventional GWAS were significantly associated with risk of BCC as groups. Further pathway analyses that integrate more skin eSNPs and/or other functional variants are warranted to verify our findings, and additional biological studies are needed to better elucidate the roles of these genes and pathways in the etiology of BCC.

## MATERIALS AND METHODS

### Study populations

A BCC GWAS has been established within the sub-cohort of participants who provided a blood sample in Harvard cohorts. Eight case-control studies nested within the Nurses' Health Study (NHS), the Nurses' Health Study II (NHS2), and the Health Professionals Follow-up Study (HPFS) were included in the current BCC GWAS: the postmenopausal invasive breast cancer case-control study nested within the NHS (BC_NHS), the type 2 diabetes case-control studies nested within the NHS and the HPFS (T2D_NHS & T2D_HPFS), the coronary heart disease case-control studies nested within the NHS and the HPFS (CHD_NHS & CHD_HPFS), and the kidney stone case-control studies nested within the NHS, the NHS2, and the HPFS (KS_NHS, KS_NHS2 & KS_HPFS). See Supplementary Material for more detailed descriptions of NHS, NHS2, HPFS, and the eight nested case-control studies. The study protocol was approved by the Institutional Review Boards of Brigham and Women's Hospital and the Harvard T.H. Chan School of Public Health.

### Inclusion and exclusion

BCC cases who had other common cancers before diagnosis of BCC were excluded. Eligible controls were free of BCC and other common cancers. According to the National Cancer Institute and the American Cancer Society, common cancers include melanoma, SCC, breast cancer, endometrial cancer, ovarian cancer, colorectal cancer, bladder cancer, lung cancer, pancreatic cancer, kidney (renal cell) carcinoma, leukemia, non-Hodgkin lymphoma, thyroid cancer, and oral cancer. Participants with identical genetic information but different cohort IDs were removed; participants whose data appeared in more than one of the eight case-control studies were included only once. In total, the BCC GWAS comprised 2,323 BCC cases and 7,275 controls of European ancestry in the United States.

### Genotyping, quality control (QC), and imputation

Samples from BC_NHS were genotyped using Illumina HumanHap550 array as part of the National Cancer Institute's Cancer Genetic Markers of Susceptibility (CGEMS) Project [41]. We used Affymetrix 6.0 arrays for the T2D_NHS, T2D_HPFS, CHD_NHS, and CHD_HPFS, and Illumina 610Q for the KS_NHS, KS_NHS2, and KS_HPFS. Quality control on SNP completion rate, sample completion rate, deviation from Hardy–Weinberg equilibrium (HWE), Mendelian consistency, minor allele frequency, and duplication samples were conducted within each study, although the thresholds were chosen slightly differently. Within each of the eight studies, we used the MACH program [42] to impute genotypes for more than 2.5 million markers, using haplotype information in the HapMap phase II data build 36(CEU) as a reference panel.

### BCC ascertainment

Disease follow-up procedures are identical for NHS, NHS2, and HPFS. Self-reported BCC case-control status is updated every two years without further pathological confirmation, however its validity has been assessed in previous studies [12, 39, 40]. The latest update was made in 2008 for the current analysis.

### Multiple tissue human expression resource (MuTHER) project and eSNPs in skin tissue

A detailed description has been published previously [28]. Briefly, the MuTHER project included 856 female individuals of European ancestry recruited from the TwinsUK Adult twin registry [43]. Skin tissues were obtained from a photo-protected area adjacent to the umbilicus by punch biopsies. RNA from skin samples was extracted using TRIzol Reagent (Invitrogen), followed by RNA quality assessment and concentration measurement. Illumina Human Ht-12 V3 BeadChip (48,804 probes) was used for expression profiling of each sample, with either two or three technical replicates. After quality control, expression profiling of skin tissue was performed on 705 individuals, and 23,596 probes were kept for further analysis. The TwinsUK study was genotyped by a combination of Illumina HumanHap300, HumanHap610Q, 1M-Suo, and 1.2M Duo 1M chips. Genetic imputation was carried out using IMPUTE software package and two reference panels: P0 [HapMap 2, release 22, combined Utah residents of Northern and Western European ancestry (CEU), Yoruba from Ibadan, Nigeria (YRI) and Asian (ASN) panels] and P1 (610k+, including the combined HumanHap610k and 1M arrays). Association of expression levels with probabilities of imputed genotypes were tested using a two-step mixed model-based score test [44, 45] and implemented in the GenABEL/ProbABEL package [46, 47] for 2,029,988 SNPs with MAF of >5% and IMPUTE info value of >0.8. In total, 667 skin samples that had both expression profiles and imputed genotypes were included in the analysis. Results of testing associations between gene expression level and SNPs were published and made publicly accessible on MuTHER's website in 2012 (http://www.muther.ac.uk/Data.html). In their study, eSNPs were called with a false discovery rate (FDR) of 1%, which corresponds to *P-value* < 3.8 × 10^−5^ [28]. We specified three thresholds (10^−5^, 5×10^−5^, and 10^−4^) around this p-value for eSNPs selection in the current study. We used the significance level of 10^−5^ in main analysis and the other two in sensitivity analysis.

### Statistical analysis

#### Association analysis

We used a multivariate logistic regression model, adjusted for age, and the first three principal components of genetic variation, to evaluate the associations between eSNPs and BCC risk in each of the eight nested case-control studies. The principal components were calculated for all individuals on the basis of ca. 10,000 unlinked markers using the EIGENSTRAT software [48]. The within-study association results for each of the eSNPs were combined by implementing inverse variance-weighted meta-analyses in METAL software [49].

#### eSNP enrichment analysis

We integrated the eSNP information into pathway-based GWAS analysis using the method of Zhong *et al.* [23]. For a gene whose expression is associated with multiple eSNPs, we chose the eSNP that had the most significant association with BCC risk as this gene's representative. Then we assigned these genes into the pathway defined by pathway databases. We evaluated the association of each pathway with risk of BCC with an Enrichment Score (ES), which was calculated from the weighted Kolmogorov-Smirnov-like running-sum statistic. This ES reflects the overrepresentation of genes within this pathway at the top of the entire ranked list of genes being tested. We permuted the case-control status and re-calculated the statistic values 1,000 times to assess the significance of each ES. To allow direct comparison of pathways of different sizes, a normalized enrichment score (NHS) was computed for each pathway. The FDR was calculated to estimate the proportion of false positive findings by using NES [50]. We set the significance level for the pathway analysis as p-value < 0.05 and FDR < 0.2.

#### Pathway databases

We used human biological pathways as defined in the Kyoto Encyclopedia of Genes and Genomes (KEGG, http://www.genome.jp/kegg/pathway.html/) database [51] as the primary pathway collection. Gene Ontology (GO, http://geneontology.org/) and BioCarta (http://www.biocarta.com/) databases were also included as secondary pathway collections. All pathways that contain at least 3 but at most 200 genes represented by eSNPs were tested.

### Sensitivity analysis

Results (p-values) of all tested SNP-gene expression pairs are published on the MuTHER website. The threshold to identify SNPs that are significantly associated with at least one gene's expression in skin tissue is arbitrary. As the threshold becomes less stringent, the number of genes that can be represented by eSNPs increases and the surrogate eSNP for a particular gene may change. Therefore, we changed our threshold for eSNP selection to 5×10^−5^ and 10^−4^ respectively for the purpose of sensitivity analysis.

## SUPPLEMENTARY MATERIALS TABLE


