# Supplementary material for: Pathway analysis of expression-related SNPs on genome-wide association study of basal cell carcinoma
Source: Oncotarget. 2016 May 6;7(24):36885–95. doi: 10.18632/oncotarget.9212 (PMC5095046; doi:10.18632/oncotarget.9212)
Supplement: Supplementary file 1 [file oncotarget-07-36885-s001.pdf]

SUPPLEMENTARY MATERIALS

Detailed description of study population

Nurses' health study (NHS)

The NHS is a prospective cohort study established in 1976 with 121,700 female U.S registered nurses, who were then 30-55 years old. All of them completed and returned a mailed self-administered questionnaire about their medical histories and lifestyle at the baseline. In 1989 and 1990, a total of 32,826 women provided blood samples. Information regarding medical history, lifestyle, and disease diagnoses was updated every 2 years with a follow-up rate of 90%.

Health professionals follow-up study (HPFS)

The HPFS began in 1986 with 51,529 U.S. male health professionals who were 40-75 years old at initial recruitment. They all answered a detailed mailed questionnaire at the inception of the study. Disease- and health-related information was obtained and updated through biennial questionnaires. Between 1993 and 1994, 18,159 of these men provided a blood sample. The average follow-up rate for this cohort over 10 years is greater than 90%.

Nurses' health study II (NHS2)

The NHS2 was established in 1989, when 116,671 female registered nurses aged 25–42 and residing in the United States at the time of enrollment responded to an initial questionnaire on their medical histories and baseline health-related exposures. Information regarding medical history, lifestyle risk factors, and disease diagnoses was updated every 2 years with a follow-up rate of above 90%. Blood samples from 29,616 nurses were collected in the late 1990's.

BCC GWAS set

A BCC GWAS set has been established within the sub-cohort of participants who provided a blood sample. Eight case-control studies were included in current BCC GWAS, they are:

1. Postmenopausal invasive breast cancer case-control study nested within the NHS (BC_NHS): Eligible cases in this study consisted of women with pathologically confirmed incident breast cancer from the subcohort who gave a blood specimen. Cases with a diagnosis after blood collection up to June 1, 2000 with no previously diagnosed cancer except for non-melanoma skin cancer were included. One control for each case was randomly selected among women who gave a blood sample and were free of diagnosed cancer (excluding non-melanoma skin cancer) up to and including the interval in which the case was diagnosed. Controls were matched to cases on year of birth, menopausal status, recent post-menopausal hormone (PMH) use, month of blood return, time of day of blood collection, and fasting status at blood draw [1].
2. Type 2 diabetes (T2D) case-control study nested within the NHS and HPFS (T2D_NHS and T2D_HPFS): Diabetes cases were defined as self-reported incident diabetes confirmed by a validated supplementary questionnaire. For cases before 1998, diagnosis was made using criteria consistent with those proposed by the National Diabetes Data Group (NDDG). For cases during the 1998 and 2000 cycles, the American Diabetes Association's diagnostic criteria were used for the diagnosis of diabetes cases. The nondiabetic control subjects were matched to cases on age, month and year of blood draw, and fasting status [2].
3. Coronary heart disease (CHD) case-control study nested within the NHS and HPFS (CHD_NHS and CHD_HPFS): In both the NHS and HPFS, participants who had reported an incident CHD event on the follow-up questionnaire were contacted for confirmation and permission to review medical records was requested. Medical records for deceased participants were also sought for deaths that were identified by families and postal officials and through the National Death Index. Physicians blinded to the participant's questionnaire reports reviewed all medical records. Fatal CHD cases were identified primarily through review of medical records [3]. Among participants who provided blood samples and who were without cardiovascular disease or cancer at blood draw, incident CHD cases occurring after blood draw were selected as cases. Controls were selected in a 2:1 ratio matched to cases on age, smoking, and month of blood return.
4. Kidney stone case-control study nested within the NHS, NHS2 and HPFS (KS_NHS, KS_NHS2 and KS_HPFS): Participants from KS_NHS, KS-NHS2 and KS_HPFS were individuals who performed a 24-hour urine collection; two-thirds had a history of incident nephrolithiasis. Details regarding the urine collection [4] and the confirmation of kidney stone disease were published previously [5]. The biennial questionnaires have asked whether a participant had been diagnosed with kidney stone. For newly reported cases, an additional questionnaire was sent to inquire date of occurrence and symptoms. Studies have been conducted to confirm the validity of the self-reported stones [6, 7]. A control was randomly selected from the blood cohorts of NHS, NHS2, and HPFS for each case, matching on age, time of blood draw, and fasting status.

REFERENCES

1. Hunter, D.J., et al., A genome-wide association study identifies alleles in FGFR2 associated with risk of sporadic postmenopausal breast cancer. Nat Genet, 2007. 39: p. 870-4.
2. Qi, L., et al., Genetic variants at 2q24 are associated with susceptibility to type 2 diabetes. Hum Mol Genet, 2010. 19: p. 2706-15.
3. Rimm, E.B., et al., Prospective study of alcohol consumption and risk of coronary disease in men. Lancet, 1991. 338: p. 464-8.
4. Curhan, G., E. Taylor, 24-h uric acid excretion and the risk of kidney stones. Kidney international, 2007. 73: p. 489-496.
5. Taylor, E.N., M.J. Stampfer, G.C. Curhan, Obesity, weight gain, and the risk of kidney stones. Jama, 2005. 293: p. 455-462.
6. Curhan, G.C., et al., A prospective study of dietary calcium and other nutrients and the risk of symptomatic kidney stones. New England Journal of Medicine, 1993. 328: p. 833-838.
7. Curhan, G.C., et al., Comparison of dietary calcium with supplemental calcium and other nutrients as factors affecting the risk for kidney stones in women. Annals of Internal Medicine, 1997. 126: p. 497-504.

Supplementary Table 1: Number of BCC cases and controls # in the eight case-control studies nested in NHS, NHS2 or HPFS

Study	Number of BCC cases	Number of controls
BC_NHS	248	816
T2D_NHS	665	2162
T2D_HPFS	597	1555
CHD_NHS	253	765
CHD_HPFS	282	715
KS_NHS	99	324
KS_NHS2	58	552
KS_HPFS	121	386
Total	2323	7275

BCC cases who had diagnosis of other common cancers before diagnosis of BCC were excluded; Controls who had other cancers were excluded; participants with identical genetic information but different cohort ID were removed; participants sampled by more than one studies were included only once. Consent withdrawals were removed.
